# Enhanced mechanical properties and biocompatibility of hydroxyapatite scaffolds by magnesium and titanium oxides for bone tissue applications

**DOI:** 10.1016/j.heliyon.2024.e33847

**Published:** 2024-06-28

**Authors:** Mehdi Arab, Panteha Behboodi, Adrine Malek Khachatourian, Ali Nemati

**Affiliations:** Department of Materials Science and Engineering, Sharif University of Technology, Tehran, Iran

**Keywords:** Scaffolds, Hydroxyapatite, Titanate, Gel-cast, Biocompatibility, Agarose

## Abstract

Significant attention has been devoted to bioactive implants for bone tissue applications, particularly composite scaffolds based on hydroxyapatite (HaP). This study explores the effects of Magnesium and Titanium oxides on the characteristics of HaP-based composite (HMT) scaffolds. The ceramic nanopowders were synthesized using in situ sol-gel, and then the scaffolds were fabricated by gel-casting technique, followed by heat treatment at 1200 °C. The thermal, microstructural, and structural properties of the samples were investigated by different characterization techniques. It was observed that the formation of the MgTiO_3_ phase in the composite scaffold was likely the key factor contributing to the improved mechanical properties. Finally, to evaluate bioactivity and biodegradability, scaffolds were immersed in simulated body fluid (SBF) buffer and analyzed by Field Emission Scanning Electron Microscopy (FESEM), and the viability of human fibroblast cells was assessed using the MTT assay. The composite scaffolds containing the MgTiO_3_ phase showed greater HaP layer formation on the scaffold surface, indicating enhanced biocompatibility.

## Graphical abstract

**Figure:**
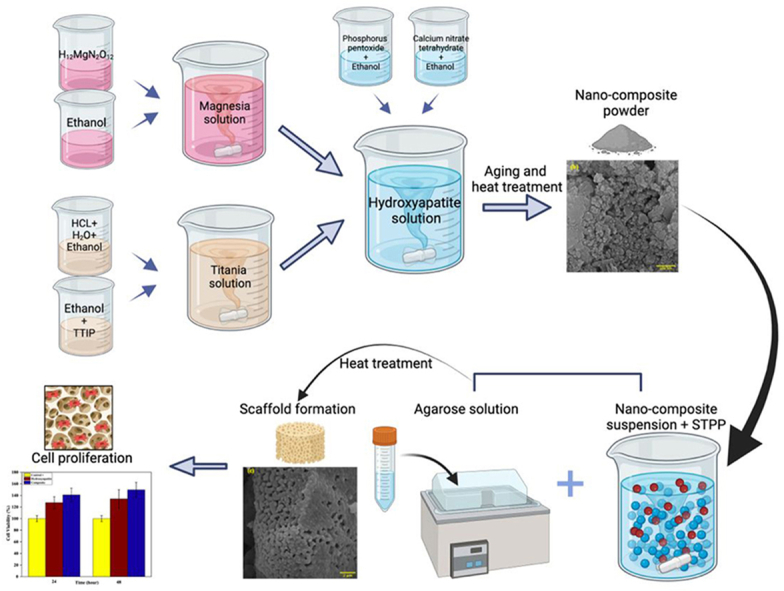

## 1 Introduction

Biomaterials are critical in improving life expectancy by providing partial or complete replacement of tissues and organs that maintain functionality and promote healing after injuries and diseases [1]. These materials fall under three categories: bioinert, bioactive, and bioresorbable, which include various materials such as metals, polymers, ceramics, and composites with broad biomedical applications [[2], [3], [4], [5], [6]]. One of the most significant applications of biomaterials is bone tissue engineering [7]. Bone, comprising organic and inorganic components, forms the skeleton of the human body. Organic bone consists of cellular compartments such as osteoblasts, osteoclasts, and stroma cells, which help in the remodeling and resorption of bone tissue. The inorganic part of bone mainly comprises calcium and phosphates, which form a scaffold to protect internal organs and enable movement [8]. The increasing number of individuals requiring bone fractures or defects that need replacement surgery each year has amplified the demand for appropriate biomaterials that mimic bone tissue [[9], [10], [11]]. Hydroxyapatite (HaP) is one of the ideal biomaterials, with excellent sorption capabilities and structural voids that can accommodate various cationic or anionic replacements. Other characteristics of HaP, including affordability, simplicity in synthesis, stability, reactivity, and biocompatibility, contribute to its appeal. Remarkably, the latter feature makes it a popular choice for medical procedures like bone repair or implants. HaP can also serve as a medication delivery and release medium because of its specific surface area and capacity to bind substances to that surface. According to numerous studies, HaP is an effective sorbent in the adsorption processes of metal ions, especially heavy metals and radioactive ions. Inorganic and organic materials can also be adsorbed [[12], [13], [14]]. Calcium and phosphate ions make up almost 90 % of the mineral phase of bone. Therefore, inorganic bioceramics such as tricalcium phosphate (TCP) and HaP are commonly used as leading biomaterials that resemble bone scaffolds. However, the brittle nature of inorganic bioceramics like HaP cannot meet the desired toughness, and its strength is unsuitable for clinical applications [[15], [16], [17], [18], [19]].

Improving the mechanical properties of inorganic bioceramics is crucial for their successful application. Conjugating bioceramics, such as HaP, with polymers or metals, can significantly enhance their mechanical properties. This, in turn, facilitates biological processes such as cell attachment, migration, proliferation, and differentiation, leading to bone regeneration, angiogenesis, and homeostasis. Metal oxides, including aluminium oxide, zirconium oxide, silicon oxide, and titanium oxide, have been found to have the potential to promote HaP characteristics among all bioresorbable materials. Magnesium and its alloys are particularly promising when used with HaP, as they exhibit mechanical properties similar to natural bone, including elastic modulus and density [[20], [21], [22]]. Moreover, magnesium is biodegradable, making it an excellent candidate for bone implants [[23], [24], [25], [26], [27]]. Sahmani et al. [28] reported that adding 10 wt% MgO nanoparticles to 3D-printed polycaprolactone/β-TCP scaffolds increased the elastic modulus from 0.8 MPa to 1.6 MPa and the pH from 7.4 to 8.5 after 28 days of immersion in simulated body fluid (SBF). Titanium oxide is another metal alloy/oxide used in orthopaedic implants. Titanium alloys or Titanium oxide have a lower elastic modulus than other metals, which helps reduce stress shielding. Furthermore, Titanium oxide is a non-magnetic material with high corrosion resistance that enhances the diagnosis capability through Magnetic Resonance Imaging (MRI) scanning while lasting long in physiological environments [[29], [30], [31]]. Sungur et al. [32] show that adding TiO_2_ nanoparticles can increase the apatite formation rate from 0.8 mg/g for pure HaP to 1.6 mg/g for HaP-10 wt% TiO_2_. Another study reported that adding 1.5 wt% of TiO_2_ to ZTA-CeO_2_ ceramic composite increased the fracture toughness from 6.4 MPa M^1/2^ for pure ZTA-CeO_2_ to 7.54 MPa M^1/2^ for TiO_2_/ZTA-CeO_2_ composite, indicating an improved resistance to crack propagation [33].

Several scaffold fabrication techniques include sol-gel, gel-casting, connection/network, phase separation, extrusion, injection molding, 3D printing, freeze drying, and polymer-derived ceramic method [[34], [35], [36], [37], [38]]. Among these methods, gel-casting is a promising technique for fabricating porous ceramics. Gel-casting can produce HaP scaffolds with high porosity (up to 80 %) and good mechanical strength (up to 20 MPa). These scaffolds can be used for bone defect repair, drug delivery, and tissue engineering applications [39,40]. Agarose is a natural polymer extracted from seaweed that can be used as a gelling agent in the gel-cast method. Agarose has some advantages for this purpose, such as easy casting, low charge density, suitability for DNA separation by electrophoresis, nontoxicity, biocompatibility, and ability to form a gel matrix for chromatography [[41], [42], [43]].

The present study describes the synthesis of nanocomposite powders based on HaP through the in situ sol-gel method. Then, scaffolds were fabricated from synthesized powders using a gel-casting technique and agarose as a gelling agent. The effect of Magnesium and Titanium oxide additives on the characteristics of the synthesized nanocomposite powders and the HaP-based composite scaffolds are also investigated. This approach aims to improve the scaffolds’ mechanical properties and biocompatibility. Previous studies have shown that adding metal oxides like MgO and TiO_2_ can significantly enhance the mechanical properties of HaP [[16], [17], [44]]. Moreover, the gel-casting technique is used to create the scaffolds. This fabrication method is promising for producing porous ceramics with high porosity and good mechanical strength, making them suitable for bone tissue engineering applications. Moreover, the biocompatibility and bioactivity of the scaffolds were assessed by conducting an MTT assay and submerging the scaffold in SBF, respectively. Results of the study demonstrate that Magnesium and Titanium oxide resulted in the formation of MgTiO_3_ on the surface of the scaffold, which enhanced the biocompatibility of the HaP scaffold. The study concludes that the synthesized nanocomposite powders and the fabricated scaffolds are promising for biomedical applications.

## 2 Experimental procedure

### 2.1 Materials

The synthesis of nanocomposite powder involves the use of specific chemical precursors. Calcium nitrate tetrahydrate (CaH_8_N_2_O_10_, CAS No.13477-34-4) and Phosphorus pentoxide (P_2_O_5_, CAS No.1314-56-3) serve as precursors for HaP, while Titanium isopropoxide (C_12_H_28_O_4_Ti, CAS No. 546-68-9) functions as a precursor for Titania. Magnesium nitrate hexahydrate (H_12_MgN_2_O_12_, CAS No. 13446-18-9) is a Magnesia precursor. In addition, ammonia (NH_3_, CAS No. 7664-41-7) and hydrochloric acid (HCl, CAS No. 7647-01-0) are utilized to adjust pH levels, while ethanol (C_2_H_6_O, CAS No. 64-17-5) and deionized (DI) water are used as solvents. Agarose is employed as a gelling agent for the gel-casting method, while sodium tripolyphosphate (STPP, CAS No. 7758-29-4) is utilized as a dispersing agent. All chemical reagents were acquired from Merck Co. and used without purification or further treatment.

### 2.2 HaP and HMT composite powders synthesis

Nanocomposite powders (Hydroxyapatite/Magnesia/Titania (HMT) composite powders) were synthesized through a situ sol-gel process at room temperature (RT) and then calcined at 700 °C for 1 h. Fig. S1 illustrates the initial substances, their chemical composition, and the corresponding vendor for composite sol formation. In summary, the desired amount of Calcium nitrate tetrahydrate and Phosphorus pentoxide were mixed with a ratio of Ca/P = 1.67. Ammonia was added during the mixing process to maintain the pH at 8. Subsequently, to make the composite sol, 15 wt% of Titania and Magnesia sols with a ratio of Mg/Ti = 2 were added to the solution, followed by 4 h of stirring. Afterwards, the sol was kept at RT for an ageing process for 48 h. Finally, the sol was dried at 40 °C in an oven and then heat-treated to 700 °C at 10 °C/min and 1 h soaking time. HaP powders were also synthesized using the same procedure without adding titanium or magnesium sols during the in-situ sol-gel process. In the case of HaP powders, the calcination temperature was 600, 700, 800, and 900 °C, and based on thermal and structural analysis, 700 °C was selected as the optimum temperature for powder calcination.

### 2.3 Scaffolds fabrication

Using gel-casting techniques, HaP and HMT composite scaffolds were fabricated. First, half of the HaP powder and the HMT composite powder are gradually dispersed in 10 mL DI water. To ensure a homogenous suspension, Sodium Tripolyphosphate (STPP) is added to the suspension as a dispersing agent at a weight ratio of 1/30. This weight ratio is the optimum ratio to obtain a homogenous suspension. The STPP molecules attach to the surface of the particles and create a repulsive force between them, which helps prevent agglomeration and promotes even distribution of the particles in the suspension. Next, the remaining powder is added to the suspension, and the mixture is stirred for 1–3 h to ensure a homogenous suspension is achieved. Separately, a 4 wt% agarose solution is prepared in a water bath at 90 °C–95 °C for 15 min. The agarose solution and composite suspension are mixed and stirred for 10 min to obtain a homogenous suspension. The final suspension is poured into prepared molds with a radius of 15 mm and a height of 30 mm. Then, the molds are dried in the oven at 35 °C for 15 h, allowing the samples to solidify. Once the samples have solidified, they are sintered in an electrical furnace. The sample is first heated up to 600 °C slowly at a 1 °C/min rate to remove agarose at this temperature. Then, the heating rate is increased to 2 °C/min up to 1200 °C with a soaking time of 1 h to complete the heat treatment process. This process ensures that the scaffolds are fully formed and hardened, creating a strong and durable structure [45].

### 2.4 Structural, microstructural, and physical characterizations

Simultaneous thermal analysis (STA) was determined in the range of 25 °C–1100 °C with a heating rate of 10 °C/min to investigate the thermal behavior of samples (STA 504 BAHR Company, Germany). The Fourier Transformed Infrared Spectroscopy (FTIR) was conducted in the 400 - 4000 cm^−1^ range (1.92 cm^−1^ measurement step) using the Spectrum RX1 Fourier Transform Infrared Spectrometer device by PerkinElmer USA. The microstructural analysis was performed by Field Emission Scanning Electron Microscopy (FESEM) (MIRA3, TESCAN Technology Company, Czech Republic). ImageJ software is used to measure pore and particle size and size distribution. The phase characteristics of the samples were evaluated by X-ray diffraction (XRD) analysis using an Xpert-Philips Diffractometer equipped with Cu Kα radiation (40 kV, 30 mA, 2θ range between 20 and 80°, 0.05° step size, scan speed of 3°/min).

The compressive strength of the scaffolds was evaluated using a Hounsfield (H10KS) machine at a speed of 0.6 mm/min. The jaws consist of two steel cylinders with a cross-sectional diameter of 4 cm. Cylindrical samples 10 mm in height and 12 mm in diameter were prepared for mechanical measurement. An S-type load cell with a capacity of 10 kN force is used. The test is repeated for five samples. Archimedes’ method has been used to determine the porosity of the scaffolds. In this method, according to Equation (1), the density of the bulk samples could be measured by measuring the dry weight (W_1_), the weight of the oil-impregnated samples (W_2_), and the weight of the oil-impregnated samples suspended in water (W_3_) [46].

(1) ρpellet=w1.ρwaterw2−w3

### 2.5 Bioactivity evaluation

The samples were placed in the SBF at physiological pH 7 and at 36.5 °C to assess the bioactivity of the HaP and HMT scaffolds. The SBF was created following published guidelines [47,48]. The samples were immersed in SBF for 7 days without replenishing the solution, and then they underwent filtering, DI water rinsing, and RT drying. During this time, a pH electrolyte was used daily to check the solution's pH. Using FESEM micrographs, the development of the HaP layer on the samples was confirmed.

### 2.6 Cytotoxicity measurement (MTT assay)

To assess the viability and cytotoxicity of human cells on HaP and HMT scaffolds, MTT assay according to the manufacturer's protocol (Sigma Chemical Co.) and PMCID was used [49]. Briefly, human fibroblast cells were seeded on HaP and HMT scaffolds in a 24-flat bottom wall plate with a density of 105 per well. The cells were cultured for 24 and 48 h in a 37 °C incubator with 5 % CO_2_. To initiate the MTT assay, cell culture medium was aspirated, followed by adding 100 ***μ***L MTT solution (5 mg/mL in PBS) (Sigma Chemical Co.) and 900 ***μ***L of DMEM without serum to each well. After 4 h of incubation, the serum-free cell culture medium with MTT solution was aspirated. The formazan crystals were solubilized with an acidified isopropanol solution (0.04 N HCl). Finally, the viability of the cells was obtained as a function of absorbance determined by spectrophotometry at a 570 nm wavelength using an ELISA plate reader (BIO-RAD, model 3550-UV, Hercules, CA, USA). The mean value of three aliquots was used for each well. The results of the MTT assay were normalized to control well.

## 3 Results and discussion

### 3.1 HaP and HMT composite powders

**Fig. 1:**
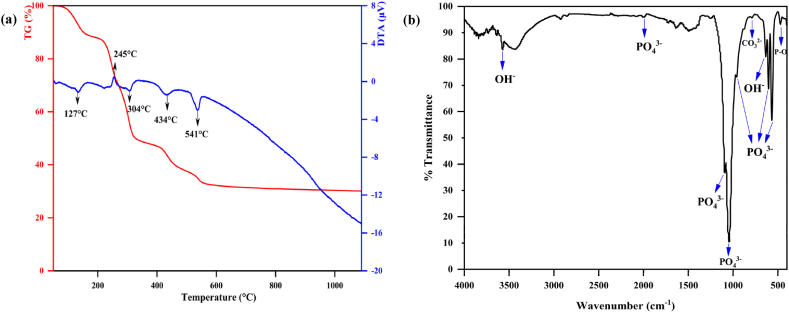
(a) STA analysis of synthesized HaP powder and (b) FTIR spectrum of HaP powder calcined at 700 °C.

The thermal behavior of the dried gel from the sol-gel synthesis method is studied by STA analysis (Fig. 1a). In the TGA curve, the first weight loss step from RT to about 200 °C is from the removal of structural and bonded water. Then, the second weight loss step is between 200 and 400 °C, which is in accordance with the DTA peak of about 245 °C and can be related to the release of ammonia and nitrate from the precursor. The third weight loss step can be attributed to the hydrogen phosphate's ion condensation reaction at a temperature of 400 °C, and its weight loss up to a temperature of about 450 °C is evident. The peak from 450 to 600 °C at the DTA diagram is due to the calcination in this region, confirming the phase transition from the amorphous state to the calcium phosphate crystal. The DTA curve does not show any significant peak in the higher temperature region, indicating the thermal stability of the HaP phase [[50], [51], [52]].

The FTIR spectra of the synthesized HaP powder calcined at 700 °C are presented in Fig. 1b. The assignment of the vibration bands, according to the literature data, is summarized in Table S1. P–O vibration modes are observed in 2002 cm^−1^, 1091 cm^−1^, 1041 cm^−1^, 961 cm^−1^, 603 cm^−1^ and 567 cm^−1^, and the O–H peak is also determined at 3570 cm^−1^ and 629 cm^−1^. Rodrigguez et al. [53] consider the peak for HaP to be 3450-3650 cm^−1^. The 900-11100 cm^−1^ peak is related to (PO_4_)^3-^. (PO_4_)^3-^ tensile and flexural modes are between 560 and 600 cm^−1^. Also, carbonate groups are observed in 873 cm^−1^ and 2900 cm^−1^ [54].

**Fig. 2:**
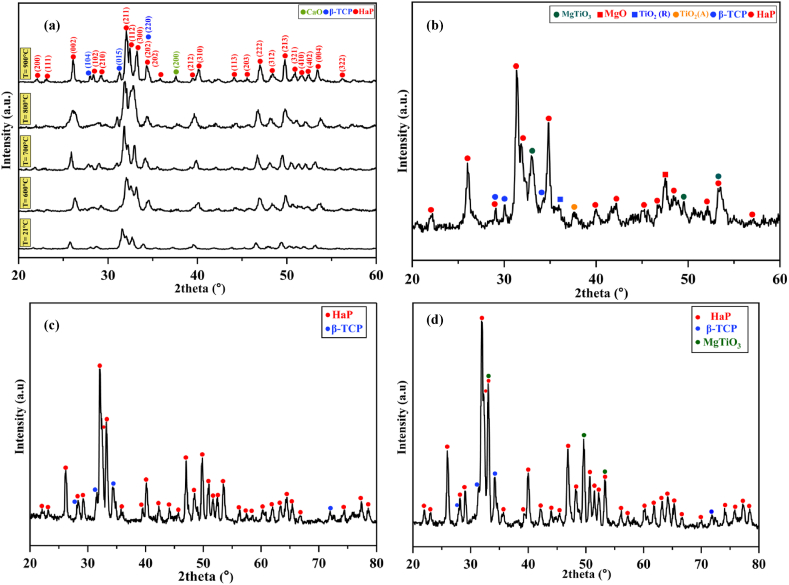
XRD patterns of (a) HaP powders calcined at various temperatures, (b) HMT composite powders calcined at 700 °C, (c) HaP scaffold sintered at 1200 °C, and (d) HMT composite scaffold sintered at 1200 °C.

XRD analysis of synthesized and calcined HaP powders at 600, 700, 800, and 900 °C temperatures are shown in Fig. 2a. The HaP phase is present at all temperatures. According to JCPDS 46–0905, the main peaks at 25.88°, 31.76°, 32.16°, 32.92°, and 39.8° correspond to the characteristic peaks of (002), (211), (112), (300), and (310) of the HaP phase, respectively. The intensity of the HaP peaks and the crystallinity of the powders have increased with increasing temperature. At higher temperatures range of 800–900 °C, the peak of CaO is observed. However, at 600 and 700 °C, the peak CaO is almost negligible and has not been detected. According to some studies, the CaO impurity phase with the HaP phase is inappropriate since it can make the material more susceptible to degradation in biological environments. CaO can also negatively affect the biological milieu, causing inflammation or infection. Moreover, CaO can alter the stoichiometry and crystallinity of HaP, which may affect its mechanical and biological properties. Therefore, removing or reducing CaO and other impurity phases from HaP is essential to ensure its quality and performance [[55], [56]].

The β-TCP (JCPDS number: 09–0169) phase at 700, 800, and 900 °C is also detected in the XRD patterns due to the partial decomposition of HaP [[57], [58]]. According to some studies [59], the β-TCP phase can be formed as an intermediate phase during the synthesis of HaP through various methods, including sol-gel synthesis. The formation of β-TCP during HaP synthesis occurs through different mechanisms, including the decomposition of HaP at high temperatures. At 700 °C, the decomposition of HaP can release water and hydroxyl ions, which can react with calcium ions to form β-TCP. The reaction can be described as follows:

(2) 3Ca_10_(PO_4_)_6_(OH)_2_ → 2Ca_3_(PO_4_)_2_ + CaO + 5H_2_O

The CaO produced in the reaction can further react with phosphoric acid to form β-TCP:

(3) CaO + H_3_PO_4_ → Ca_3_(PO4)_2_ + H_2_O

Therefore, the formation of β-TCP during HaP synthesis at 700 °C can be due to the decomposition of HaP, which can release calcium ions that can react with phosphate ions to form β-TCP [[57], [58], [60]]. Finally, HaP powders with the β-TCP phase have slightly better degradation resistance than pure HaP. The absence of CaO also enhances their mechanical strength. Therefore, the optimum calcination temperature for synthesizing a desirable HaP powder for bioapplication can be produced by calcining at 700 °C without the CaO phase and with the β-TCP phase.

Fig. 2b shows the XRD analysis of the composite powders. As shown in Fig. 2b, the sample synthesized at 700 °C has a predominant HaP phase. Moreover, the presence of the MgTiO_3_ phase (JCPDS number: 02–901) is confirmed along with small amounts of TiO_2_ and MgO phases after heat treatment at 700 °C. This composite powder is named HMT (Hydroxyapatite - Magnesium Titanate) hereafter. At 700 °C, the reaction between MgO and TiO_2_ is thermodynamically favorable due to the negative Gibbs free energy of the formation of MgTiO_3_. This means that MgTiO_3_ is more stable than the individual MgO and TiO_2_ phases and, therefore, will tend to form when the two precursors are heated together. The formation of MgTiO_3_ can also be facilitated by the presence of HaP in the composite particles. HaP, a ceramic material with a complex crystal structure, can provide nucleation sites for forming MgTiO_3_. It allows the reaction between MgO and TiO_2_ to occur more readily, leading to a higher yield of MgTiO_3_. The reaction of MgO and TiO_2_ at 700 °C has resulted in the formation of MgTiO_3_, as shown in Eq. (4) [[61], [62]].

(4) MgO + TiO_2_ → MgTiO_3_ ΔG_973K_ = 66.668 kJ, ΔH_973K_ = −102.061 kJ

**Fig. 3:**
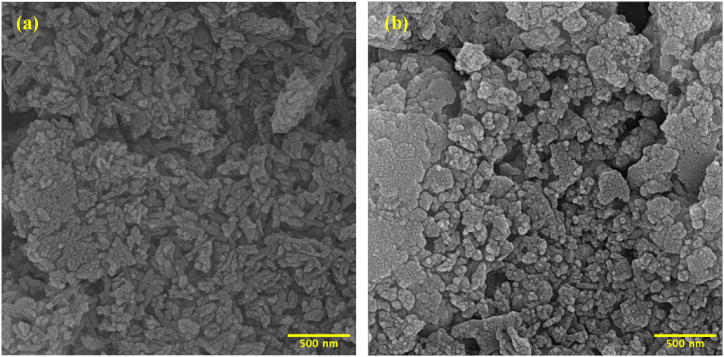
FESEM Images of (a) HaP powders calcined at 700 °C and (b) HMT composite powders calcined at 700 °C.

Fig. 3a and b shows the FESEM micrographs of HaP and HMT nanopowders, respectively. The HaP average particle size is about 50 nm, while the HMT composite powder average particle size is about 30 nm. The morphology of synthesized HaP powders is elongated, while the morphology of composite powders is more spherical. However, agglomerates of nanoparticles can be seen in these images. The Elemental mapping images of HMT nanocomposite powders are shown in Fig. S2. The presence of the main elements of Ca, P, O, and Mg from the HaP and β-TCP phases and Mg and Ti elements from composite phases with homogenous distribution is confirmed.

### 3.2 HaP and HMT composite scaffolds

The scaffolds are fabricated using the gel-casting technique using the HaP powder and HMT composite powders calcined at 700 °C. Fig. 2c and d show the XRD pattern of the HaP and HMT composite scaffolds sintered at 1200 °C, respectively. According to Fig. 2c, more β-TCP phases are observed than in Fig. 2a, indicating that some HaP phases decompose into the β-TCP phase at high temperatures. According to Fig. 2d, the HMT composite scaffold consists of three phases: HaP, β-TCP, and MgTiO_3_. The HaP is the dominant phase (According to JCPDS 46–0905, the main peaks of HaP are 25.88°, 31.76°, 32.16°, 32.92°, and 39.8°).

**Fig. 4:**
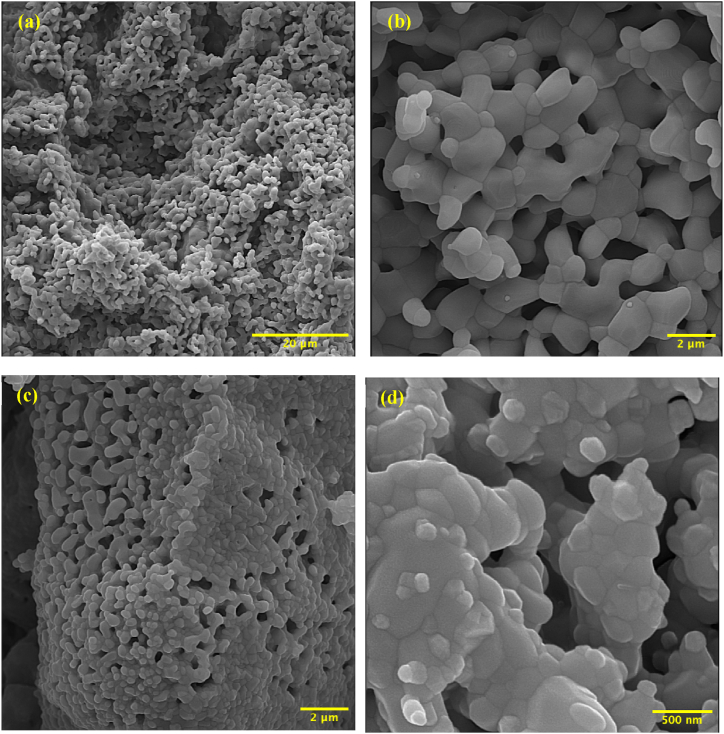
FESEM Images of (a,b) HaP scaffold sintered at 1200 °C, and (c,d) HMT composite scaffold sintered at 1200 °C.

Fig. 4a and b displays FESEM images of the HaP scaffold, which was created using the gel-casting method and sintered at 1200 °C. These images are indicative of a well-designed and interconnected pore structure. This structure is crucial for promoting cell migration and tissue ingrowth, allowing for the diffusion of nutrients and waste products throughout the scaffold and enabling the metabolic activity of cells. After measuring 50 random pores with ImageJ software, the average pore size was 500 nm ± 70 nm. The pore size range is between 50 nm and 2 μm, ideal for supporting cell attachment and proliferation and providing mechanical stability [[1], [2], [3]]. It is noted that the interconnected pore structure is crucial for promoting cell migration, tissue ingrowth, and allowing the diffusion of nutrients and waste products, enabling cell metabolic activity. Hence, this study proves that the gel-casting method can successfully produce porous ceramic scaffolds with desirable characteristics for tissue engineering applications.

Fig. 4c and d depict the FESEM images of the HMT composite scaffold, which was also fabricated using the gel-casting method and sintered at 1200 °C. The particles in Fig. 4c and d have a nonuniform semispherical shape. The HMT composite scaffold consists of interconnected pores with an average size of 250 nm ± 65 nm, and the range of pore size is between 13 nm–1.5 μm, making it suitable for biomedical applications. Additionally, the large pores in this scaffold provide a potential for tissue growth, while the smaller pores can increase biocompatibility activity and release ionic products. It is commonly believed that pore sizes of at least 100 μm are necessary for optimal bone cell growth and osteoconduction. However, the specific context and material properties of the scaffold must also be considered. The 500 nm pore size in our HaP scaffold is at the lower end of the spectrum for promoting cell attachment and proliferation. Nonetheless, it plays a crucial role in maintaining the mechanical integrity and strength of the scaffold. Smaller pore sizes can significantly enhance the scaffold's structural stability, which is essential for its practical application in load-bearing sites [63]. Research suggests that smaller pores can improve the mechanical strength of scaffolds, making them more suitable for applications requiring high structural integrity. This is particularly important in ensuring that the scaffold can withstand physiological loads without collapsing [64]. The balance between porosity and mechanical strength is critical, and while larger pores are beneficial for cell infiltration, smaller pores can support the scaffold's overall durability. Although larger pores are typically preferred for bone cell infiltration, studies have shown that smaller pore sizes (down to the nanometer range) can still support initial cell attachment and proliferation. For instance, osteoblasts and other bone-related cells can attach to and proliferate on scaffolds with smaller pore sizes, especially when the material provides appropriate surface chemistry and topography [65,66]. Actually, recent advancements in scaffold design have focused on creating hierarchical structures that incorporate both micro and nanopores. These designs can offer the dual benefits of mechanical strength and enhanced biological performance. Scaffolds with a combination of nano-sized and micro-sized pores can facilitate cell attachment at the nano-scale while allowing cell migration and vascularization at the micro-scale [67].

**Fig. 5:**
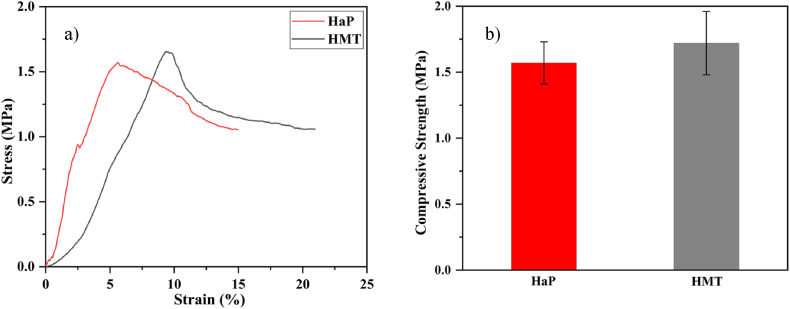
(a) Stress-Strain curve and (b) Compressive strength of the HaP and HMT scaffolds.

The stress-strain curve of the HaP and HMT scaffolds is illustrated in Fig. 5. The HaP scaffold's compressive strength is about 1.57 ± 0.16 MPa, which shows that the sample is well accepted for use in bone tissue engineering. For the HMT composite scaffold, the compressive strength is about 1.72 ± 0.24 MPa, which means that the compression strength of the sample has been improved. It is probably due to the MgTiO_3_ phase. This tough material can withstand high stress and deformation without cracking or fracturing, making it more resistant to mechanical failure under compressive loads required for bone tissue engineering applications. Furthermore, MgTiO_3_ has a crystal structure and chemical composition similar to HaP, enhancing the interfacial bonding between the two materials. This bonding increases the cohesion and adhesion of the scaffold, leading to improved mechanical properties [68]. Also, the MgO and TiO_2_ phases identified by XRD could influence the compressive strength of the HMT composite scaffold. The presence of these phases could contribute to the improved mechanical properties of the composite scaffold compared to the pure HaP scaffold. MgO has been shown to have a toughening effect on ceramics due to its ability to absorb energy and prevent crack propagation. Similarly, TiO_2_ has been reported to improve the mechanical properties of ceramics by enhancing their hardness and toughness [[69], [70]].

The Archimedes method has been used to investigate the porosity and density of the fabricated scaffolds. The average density of HaP and HMT composite scaffolds are 62 ± 4 % and 45 ± 2.5 %, respectively. It was previously reported that the Mg incorporation into HaP scaffolds reduced their porosity and increased their density. The smaller ionic radius of Mg can explain this compared to Ca, which leads to a more compact structure of HaP [71]. However, this also reduces the pore size and interconnectivity of HaP scaffolds.

### 3.3 Bioactivity of scaffolds

**Fig. 6:**
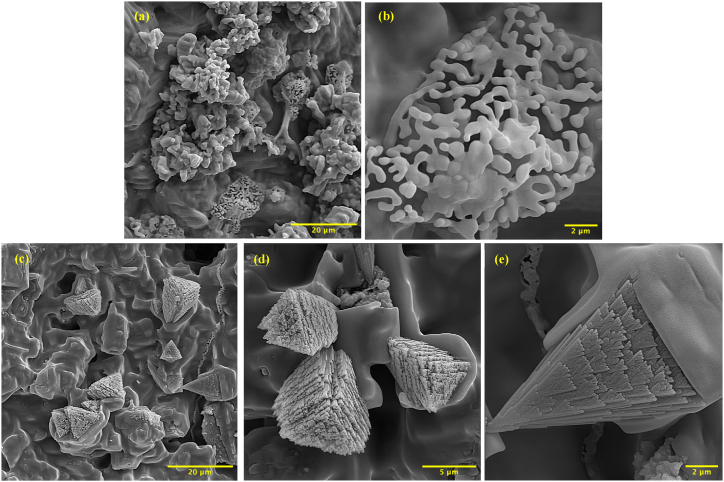
FESEM Images of (a,b) HaP scaffold immersed in SBF solution, and (c,d,e) HMT composite scaffold immersed in SBF solution.

The scaffolds were dipped in SBF solution to study their bioactivity and apatite formation on their surface. Fig. 6 (a,b) shows FESEM images of HaP scaffolds immersed in SBF solution for 72 h. The images reveal the formation of a uniform layer on the scaffold surface. During immersion, several steps occur: H_3_O^3+^ is absorbed, OH^−^ groups are generated, and positive ions like Ca^2+^ are removed. This results in a negative charge on the scaffold's surface. Ca^2+^ ions from SBF lead to calcium hydroxide production, creating a positive potential surface charge that facilitates H_3_PO^3+^ ion deposition. This process creates amorphous calcium phosphate, which forms on open pores and surfaces in contact with the SBF solution. The presence of HaP particles acts as nucleation sites for calcium phosphate. Fig. 6 (c,d,e) displays images of composite scaffolds soaked in SBF solution for 72 h, revealing triangular apatite crystals with a regular arrangement on the scaffold surface. The triangular shape of the crystals indicates their orientation, which may be related to the crystal structure of HaP. To ensure accurate assessment and cross-laboratory comparisons, following a standardized approach with a recommended ratio of 0.5 cm^2^ of exposed area per mL of SBF solution is essential. Comparing FESEM images of composite and pure HaP scaffolds immersed in SBF solution, it becomes clear that the composite scaffold exhibits a more ordered and uniform apatite formation. The less uniform and ordered apatite crystals on the pure HaP scaffold surface (Fig. 6 a,b) may be attributed to the presence of materials like MgO, TiO_2_, and β-TCP in the composite scaffold, which act as nucleation sites and promote more uniform crystal growth. Additionally, the porous structure of the composite scaffold contributes to a higher surface area, potentially enhancing uniform apatite formation.

**Fig. 7:**
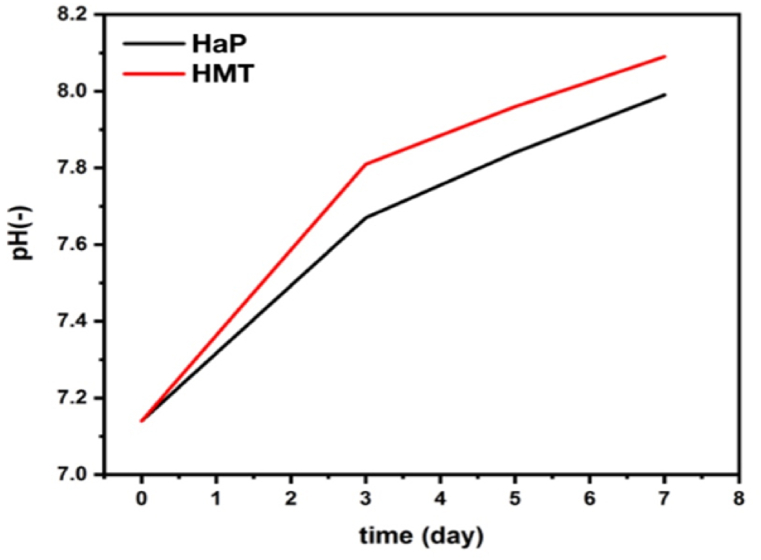
pH changes of SBF solution containing HaP and HMT composite scaffolds.

The changes in pH of a SBF solution containing HaP and HMT composite scaffolds are illustrated in Fig. 7. The exchange between H^+^ ions in the SBF solution and cations in the HaP and HMT composite samples has increased the pH of the SBF solution. The proposed exchange mechanism of Kocubo can explain the release of cations and the increase in pH to form a HaP layer on the surface [37,38]. According to the results shown in Fig. 7, an increase in the pH of the samples after seven days is due to the presence of Ca^2+^ ions and the increase of Ca^2+^ ions in the solution, which is more evident in the composite sample. This indicates that all samples, especially composite samples, are bioactive. In simpler words, the higher the dissolution rate and ion release of the HaP scaffold, the higher the pH increase in SBF. MgTiO_3_ can also increase the pH in the SBF solution by releasing Mg^2+^ ions. These ions react with H^+^ ions in the solution to form Mg(OH)_2_, which can increase the pH by consuming H^+^ ions and producing OH^−^ ions. The Mg(OH)_2_ formed can further react with Ca^2+^ ions present in the SBF solution, forming a calcium-magnesium phosphate phase on the surface of the scaffold. This phase can enhance the bioactivity of the scaffold by facilitating the formation of a stable and bioactive apatite layer on its surface. Moreover, the presence of MgTiO_3_ in the HaP scaffold can increase its dissolution rate and ion release by enhancing its solubility and reactivity. The increased dissolution rate can lead to a higher release of calcium and phosphate ions in the SBF solution, which can further react with Mg(OH)_2_ to form a calcium-magnesium phosphate phase. This process can ultimately result in a higher pH increase in the SBF solution. MgTiO_3_ can increase the dissolution rate and ion release of the HaP scaffold, leading to a higher pH increase in the SBF solution [[72], [73]]. Therefore, MgTiO_3_ can increase the dissolution rate and ion release of the HaP scaffold, which results in a higher pH increase in SBF.

### 3.4 Biocompatibility of scaffolds

**Fig. 8:**
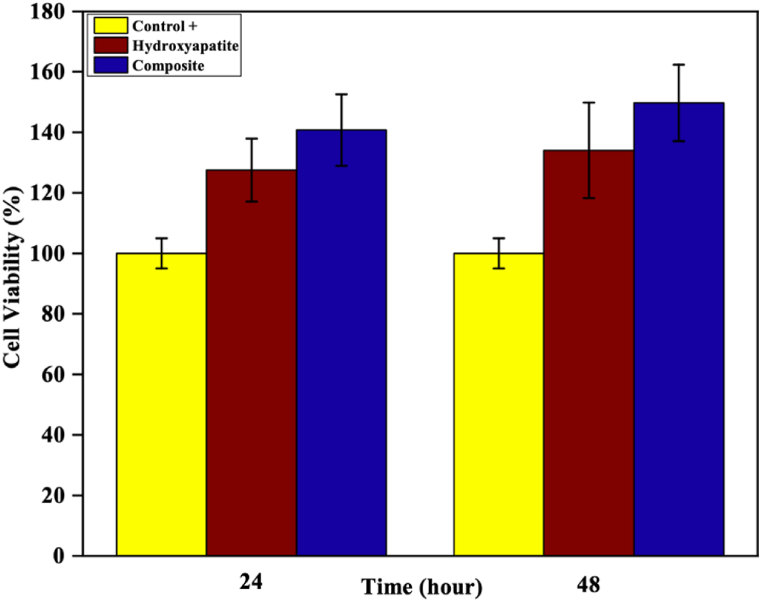
Cell viability of samples in 24 and 48 h.

The MTT assay (was performed using PC12 cells) confirmed the excellent biocompatibility of the fabricated scaffolds, as evidenced by the significant increase in cell growth within the porous sintered scaffolds, as shown in Fig. 8. The scaffolds demonstrated the potential to support cell growth and reproduction, with cell viability comparable to the control sample after 24 and 48 h of culture. Moreover, the composite scaffold exhibited superior cell viability compared to the control sample and HaP scaffold. After 24 and 48 h of incubation, the cell viability measurements indicated that the composite scaffold provided a highly conducive environment for cell attachment and reproduction, with viability percentages of 140.78 ± 11.8 % and 149.72 ± 12.62 %, respectively. Statistical analysis revealed significant differences between the HaP and Composite samples at 24 and 48 h (p-value <0.01). The use of agarose as a gelling agent in the gel-cast process notably bolstered the mechanical strength of the scaffold. Therefore, the composite shows better biocompatibility properties than HaP due to the presence of Mg and Ti ions.

## 4 Conclusion

HaP nanopowders and HMT nanocomposite powders, containing a secondary phase of MgTiO_3_, were synthesized using the in situ sol-gel method and properly calcined at 700 °C. Scaffolds were then fabricated from the synthesized nanopowders using the gel-cast technique followed by sintering at 1200 °C. The presence of the HaP phase, titanium oxide, and magnesium oxide in nanocomposite powders resulted in the formation of a secondary phase, MgTiO_3_, in composite scaffolds. This considerably improved the scaffold's mechanical strength from 1.57 ± 0.16 (pure HaP scaffold) to 1.72 ± 0.24 MPa (HMT composite scaffold). In the SBF test, HaP layers formed on the surface of both HaP and HMT composite scaffolds. For the HMT composite scaffolds, the HaP layers formed uniformly with triangle layers. An MTT assay showed 149.72 ± 12.62 % cell viability after 48 h in composite samples, indicating that the scaffolds were highly biocompatible and promoted cell growth and proliferation. Statistical analysis confirmed that the differences in cell viability between the Control +, HaP, and HMT composite samples at 24 and 48 h were significant (p-value <0.01). Using agarose as a gelling agent in the gel-cast process enhanced the mechanical strength of the scaffold. It is non-toxic nature made it an ideal choice for tissue engineering applications. Overall, the HaP-based scaffold with MgTiO_3_ secondary phase fabricated using the gel-cast method with agarose gel agent is a promising candidate for bone tissue engineering applications.

## Data availability

All data generated or analyzed during this study are included in this published article and its supplementary information files.

## Funding

No funding was received.

## CRediT authorship contribution statement

**Mehdi Arab:** Writing – original draft, Resources, Methodology, Investigation, Conceptualization. **Panteha Behboodi:** Writing – review & editing. **Adrine Malek Khachatourian:** Writing – review & editing, Supervision, Conceptualization, Project administration. **Ali Nemati:** Supervision, Project administration.

## Declaration of competing interest

The authors declare that they have no known competing financial interests or personal relationships that could have appeared to influence the work reported in this paper.
