# Supplementary material for: Enhanced mechanical properties and biocompatibility of hydroxyapatite scaffolds by magnesium and titanium oxides for bone tissue applications
Source: Heliyon. 2024 Jun 28;10(13):e33847. doi: 10.1016/j.heliyon.2024.e33847 (PMC11255589; doi:10.1016/j.heliyon.2024.e33847)
Supplement: Multimedia component 1 [file mmc1.docx]

**Supplementary Information**

**Enhanced Mechanical Properties and Biocompatibility of Hydroxyapatite Scaffolds by Magnesium and Titanium Oxides for Bone Tissue Applications**

Mehdi Arab^a^, Panteha Behboodi^a^, Adrine Malek Khachatourian^a*^, and Ali Nemati^a^

^a^ Department of Materials Science and Engineering, Sharif University of Technology, Tehran, Iran

**^*^Corresponding author:** Adrine Malek Khachatourian

**Email:** [khachatourian@sharif.edu](mailto:khachatourian@sharif.edu)


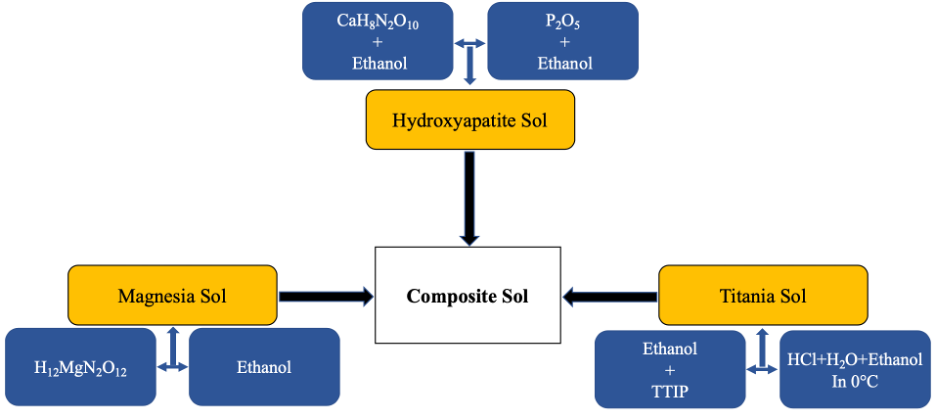


Fig. S1. Schematic of composite sol formation.


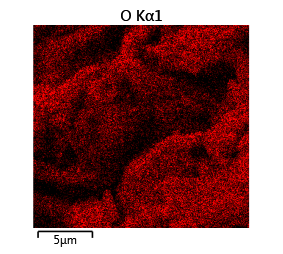

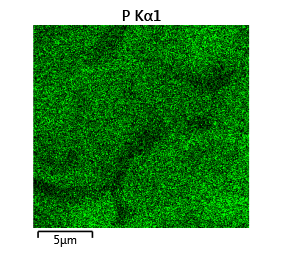

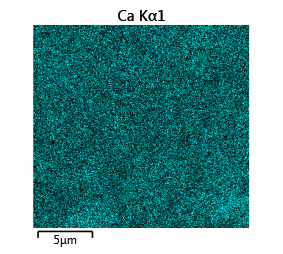

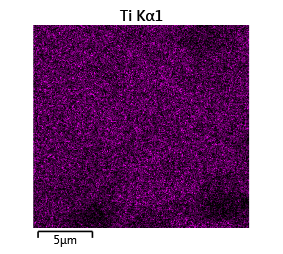

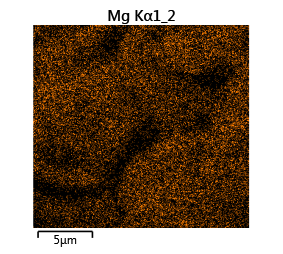

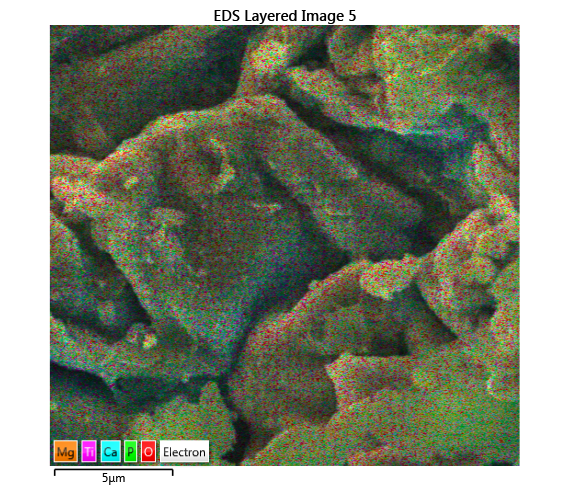


Fig. S2. Elemental mapping images of HMT composite nanopowders.

Table S1. The IR bands observed in synthesized Hydroxyapatie Powder.

| Vibrational modes | Bands at wavenumber (cm^-1^) |
| --- | --- |
| V_2_ symmetric bending mode of O–P–O in phosphate ions | 469 |
| V_4_ symmetric bending mode of O–P–O in phosphate ions | 567 |
| V_4_ symmetric bending mode of O–P–O in phosphate ions | 603 |
| symmetric stretching mode of hydroxyl ions | 629 |
| V2 of carbonate ions | 873 |
| V_1_ symmetric stretching mode of P–O in phosphate ions | 961 |
| V_3_ asymmetric stretching mode of P–O in phosphate ions | 1041 |
| V_3_ asymmetric stretching mode of P–O in phosphate ions | 1091 |
| V_1_ symmetric stretching of carbonate ions | 1321 |
| V_3_ of carbonate ions | 1417 |
| V_2_ bending mode of H–O–H in lattice H_2_O | 1630 and 1650 |
| V_3_ asymmetric stretching mode of P–O in phosphate ions | 2002 |
| V_1_ symmetric stretching mode of P–O in phosphate ions | 2072 |
| stretching mode of C – H | 2390 |
| stretching mode of C – H | 2901 |
| V_1_ stretching mode of lattice H_2_O | 3464 |
| symmetric stretching mode of hydroxyl ions | 3570 |
